# Federated Deep Reinforcement Learning-Based Task Offloading and Resource Allocation for Smart Cities in a Mobile Edge Network

**DOI:** 10.3390/s22134738

**Published:** 2022-06-23

**Authors:** Xing Chen, Guizhong Liu

**Affiliations:** School of Information and Communications Engineering, Xi’an Jiaotong University, Xi’an 710049, China; xing_chen@stu.xjtu.edu.cn

**Keywords:** smart city, mobile edge computing, task offloading, resource allocation, DDPG, federated learning

## Abstract

Mobile edge computing (MEC) has become an indispensable part of the era of the intelligent manufacturing industry 4.0. In the smart city, computation-intensive tasks can be offloaded to the MEC server or the central cloud server for execution. However, the privacy disclosure issue may arise when the raw data is migrated to other MEC servers or the central cloud server. Since federated learning has the characteristics of protecting the privacy and improving training performance, it is introduced to solve the issue. In this article, we formulate the joint optimization problem of task offloading and resource allocation to minimize the energy consumption of all Internet of Things (IoT) devices subject to delay threshold and limited resources. A two-timescale federated deep reinforcement learning algorithm based on Deep Deterministic Policy Gradient (DDPG) framework (FL-DDPG) is proposed. Simulation results show that the proposed algorithm can greatly reduce the energy consumption of all IoT devices.

## 1. Introduction

With the full development of the fifth-generation mobile communication research, the information and intelligence of cities have been greatly developed. More and more smart facilities are deployed in every corner of the city, which enhance the quality of life for citizens. In the era of IoT, smart cities power and monitor a variety of intelligent IoT devices. Accompanied by intelligent devices, IoT applications are designed, such as smart parking, smart traffic, and smart security. These applications can generate some computation-intensive tasks such as camera tracking and object recognition. In the traditional central cloud network, these tasks will be offloaded to the central cloud server for execution. However, the central cloud network faces some challenges, as follows. (1) More users are served, which is easy to cause network congestion. (2) Since the central cloud server is far away from users, the data transmission process consumes a lot of time. As a main evolution technology in the 5G, MEC provides a good direction to solve these challenges [1,2]. MEC server is deployed at the edge of the core network, which is closer to users. The computation-intensive tasks can be offloaded to the MEC server for reducing the delay, network congestion, and energy consumption of IoT devices [3,4].

Based on the above description, how to make a reasonable offloading decision and resource allocation scheme subject to limited resources has become a key problem. The joint optimization of task offloading and resource allocation is a mixed-integer nonlinear programming problem [5,6]. Currently, scholars have some research works on task offloading and resource allocation. The joint problem is solved by splitting it into several sub-problems, relaxation variables, and deep reinforcement learning based on Deep Q Network (DQN) framework [7,8,9]. However, the first two algorithms simplify the original problem and do not directly solve the joint optimization problem of task offloading and resource allocation. With the development of deep neural network, deep reinforcement learning has a good effect on solving environmental decision-making problems. However, the algorithm based on DQN framework is difficult to deal with the problem of fine-grained space or continuous space. Therefore, the deep reinforcement learning algorithm based on the DDPG framework is adopted in this article. The DDPG algorithm has a good effect in dealing with spatial continuous decision-making problems.

In order to obtain a better Quality of Experience (QoE), some research works adopted the cooperation method between MEC servers, or the unified scheduling method on the central cloud [10,11]. However, these collaborative and centralized processing algorithms do not consider the privacy and security problems in the process of data migration and processing. Therefore, many users are reluctant to upload their private raw data to other MEC servers or the central cloud server. To tackle the problem, the federated learning technology is proposed by Google [12]. It is a distributed machine learning framework, which consists of one central server and a set of clients [13,14,15]. The main idea of federated learning is to enable the data on clients to train their respective network models. Then, the parameters of clients are aggregated to update the network model on the server side. A better training model is obtained by the iteration between distribution and aggregation without sharing the raw data. Therefore, the federated learning is introduced into the joint optimization problem of this article to obtain a better optimization performance.

In this article, we focus on the joint optimization problem of task offloading and resource allocation based on privacy protection in smart city. The optimization objective is to minimize the energy consumption of all IoT devices within the delay threshold. Since the joint optimization is a mixed-integer nonlinear programming problem, it is difficult to solve it by the traditional programming algorithms. Therefore, based on the above description, a two-timescale federated deep reinforcement learning algorithm based on DDPG framework is proposed to solve the problem. The small timescale is to optimize the offload decision and the resource allocation scheme in each MEC server by training DDPG network. The large timescale is to aggregate the parameters of MEC servers in order to obtain a better training performance. The contributions of this paper can be summarized as follows:

1. We investigate the joint optimization problem of task offloading and resource allocation subject to the delay threshold and the limited resources. In the existing literature, the joint optimization problem is generally decomposed into multiple sub-problems. Therefore, a deep reinforcement learning algorithm based on DDPG framework is proposed to solve the joint problem. The DDPG is the combination of DQN and Actor-Critic (AC), which can solve the decision-making problem of continuous action space.
2. The federated learning is introduced into the deep reinforcement learning to enhance the training performance while protecting privacy and security. In terms of privacy and security, the federated learning only needs to upload the network parameters without the raw data. In terms of training performance, the federated learning is a distributed machine learning algorithm, which can obtain a better convergence.
3. Extensive numerical experiments demonstrate that our proposed algorithm has better convergence than the centralized algorithm, and obtains better performance gain than other comparison algorithms.

The rest of this article is organized as follows: Section 2 presents the system model, including task model, communication model and computation model. Section 3 presents the optimization problem and solution. Section 4 provides the simulation results and evaluates the performance of the proposed algorithm. Section 5 concludes this article.

## 2. Related Work

The concept of MEC was put forward many years ago. In 2013, the world’s first mobile edge computing platform was established by IBM and Nokia Siemens Network [16]. In 2014, the European Telecommunications Standards Institute (ETSI) proclaimed industry specifications for MEC, which was supported by IBM, Huawei, Intel, etc. Currently, most of the MEC research works focus on how to fully utilize the powerful computing and storage capacity of the MEC server to reduce delay and energy consumption of IoT devices [17]. Some popular contents are cached on the MEC server to reduce the delay and network backhaul load. Aung et al. [18] proposed a social-aware vehicular edge computing architecture that solves the content delivery problem by using some of the vehicles in the network as edge servers that can store and stream popular content to close-by end-users. The computation-intensive applications can be offloaded to the MEC server for execution [19]. Apostolopoulos et al. [20] proposed a joint problem of latency and energy minimization considering the data offloading characteristics of the end nodes. In this article, we only focus on the computing resource allocation of the MEC server.

The task offloading problem in the communication system will inevitably involve task scheduling, the allocation of computing and transmission resources [21,22]. Therefore, the problem can be easily regarded as a joint optimization problem of task offloading and resource allocation, which is a mixed-integer nonlinear programming problem. There are generally three types of algorithms to solve the problem. The first type of algorithm is to split the joint optimization problem into multiple sub-problems [7,23]. Zhao [24] formulated the joint optimization problem task offloading and resource allocation and decomposes it into three sub-problems named as offloading ratio selection, transmission power optimization, and sub-carrier and computing resource allocation. The joint optimization problem was decomposed into two-level sub-problems and solved by the iterative algorithm [25]. This type of algorithm is not a joint optimization algorithm for the original problem, and the efficiency of iterative optimization for several sub-problems is not high. The second type of algorithm is to relax the variables in the optimization problem [8]. Masoufdi [26] investigated the power minimization problem for the mobile devices by data offloading in a multi-cell multi-user Orthogonal Frequency Division Multiple Access (OFDMA) network. To solve the problem, it was converted to the convex form using variable changing, Difference of Convex (DC) approximation, adding a penalty factor, and relaxing the binary constraints. The lower bound and upper bound of the joint optimization problem were considered and the semi-definite relaxation and rounding methods were exploited to obtain the offloading decision [27]. The mixed integer nonlinear programming problem is transformed into a nonlinear programming problem by variable relaxation. Then, it is solved by iterative algorithm or genetic algorithm. Undoubtedly, the type of algorithm has a lower efficiency. The third type of algorithm is to use the deep reinforcement learning algorithm to solve the optimization problem. Li et al. [9] investigated the resource allocation scheme for vehicle-to-everything communications, and proposed the optimization problem of resource blocks allocation and vehicle transmission power allocation. A reinforcement learning based on DQN framework was designed to solve this problem. Suh et al. [28] proposed a DQN algorithm based network slicing technique to calculate the resource allocation policy, maximizing the long-term throughput while satisfying the Quality of Service (QoS) requirements in the beyond 5G systems. Since it is difficult for DQN algorithm to deal with the problem of fine-grained space or continuous space, a deep reinforcement learning algorithm based on DDPG framework is proposed to solve the joint optimization problem in this article.

To improve resource utilization and algorithm performance, some research works adopted the cooperation methods, such as Cloud-MEC, MEC-MEC, Cloud-MEC-Device. Naouri et al. [29] proposed a three-layer task offloading framework, which consisted of the device layer, cloudlet layer and cloud layer. A cloud-MEC collaborative computation offloading scheme was proposed in vehicular networks [24]. Chen et al. [30] studied an energy-efficient task offloading and resource allocation scheme for Augmented Reality (AR) in a multi-MEC collaborative system. Monia et al. [31] investigated the joint task assignment and power control problems for Device-to-Device (D2D) offloading communications with energy harvesting. A layered optimization method is proposed to solve this problem by decoupling the energy efficiency maximization problem into power allocation and offloading assignment. However, these collaborative and centralized processing algorithms do not consider the privacy and security problems in the process of data migration and processing. As a result, many users are reluctant to upload their private raw data to other MEC servers or the central cloud server. To solve this problem, federated learning is introduced in this article, which not only protects privacy but also improves the performance of the model.

## 3. System Model

**Figure 1:**
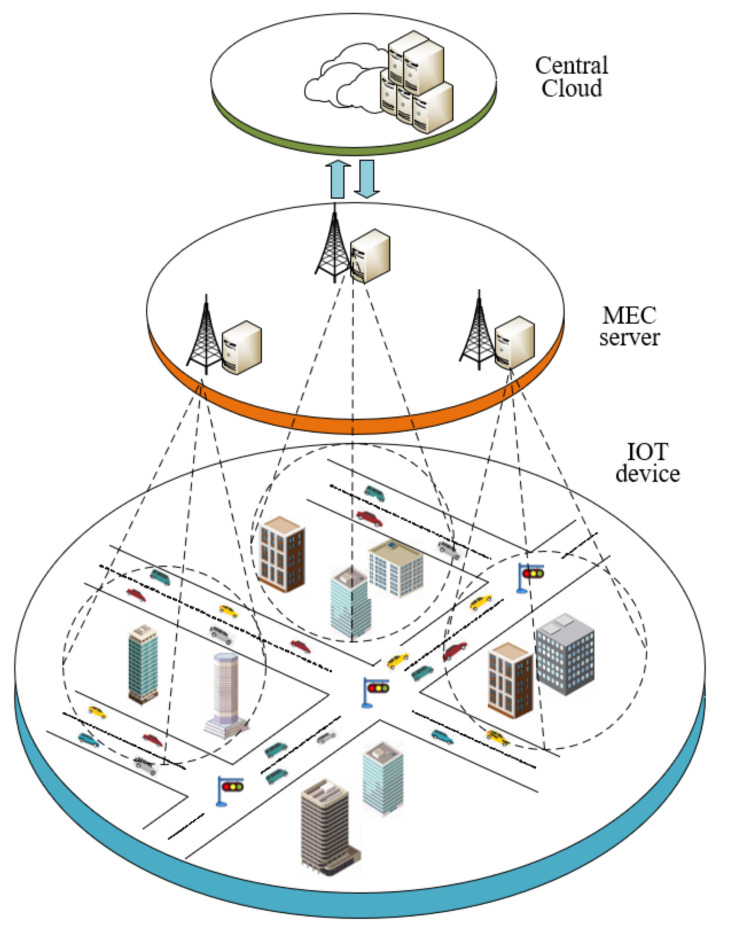
System Model.

In this article, a system model for the smart city in a mobile edge network is established, which consists of three layers: IoT device, MEC server and Central Cloud, as shown in Figure 1. The central cloud is an auxiliary role, which helps the MEC server obtain a better decision-making mechanism by aggregating the neural network parameters of each edge server. The MEC server has a powerful computing capacity, which can quickly process the tasks offloaded by IoT devices. The IoT devices can generate some tasks with strict computing requirements. Since the IoT devices have limited computing resources and limited energy, the computing tasks need to be offloaded to the MEC server for processing. In consideration of security and privacy issues, IoT devices can only offload their tasks to the trusted MEC server, not to the central cloud server. We denote the central cloud, the set of MEC servers and the set of IoT devices (the set of applications) by Γ , k∈{1,2,…,K} and n∈{1,2,…,N} , respectively. We believe that IoT devices are special devices, and each IoT device corresponds to an application. We assume that each IoT device only requests one task at the same time and the network state is constant during task processing. The specific workflow of the system is as follows. First, IoT devices generate the tasks and send the relevant information to the MEC server through the base stations at the same time. Second, a decision on offloading and resource allocation is made according to the collected task information and network status. Finally, these tasks are executed according to the offloading decision and resource allocation schemes.

### 3.1. Task Model

In the smart city scenario, there are a large number of different types of applications (such as smart security, smart traffic, smart parking, smart lamp and so on). These applications have lower real-time requirements than AR applications. Therefore, we set the delay threshold of these applications to the same, which is denoted by *T*. To describe the parametric context of each application task, we define a tuple representation as $\phi_{n}=\left(\omega_{n},\varphi_{n}\right)$ . Specifically, $\omega_{n}$ and $\varphi_{n}$ denote the data size (bit) and the computing workload (CPU cycles) of the task generated by IoT device *n*, respectively. The relationship between $\omega_{n}$ and $\varphi_{n}$ is expressed as $\varphi_{n}=\eta_{n}$ , where η_n_ denotes the computing workload per bit. In this article, the offloading decision is denoted by α_n_ ∈ {0,1}. If α_n_ = 0, the aplication task requested by IoT device *n* will not be offloaded by the edge server and will be processed on the IoT device *n*. If α_n_ = 1, the application task requested bu IoT device *n* will be offloaded to the MEC server. The important notations used in the rest of this article are summarized in Table 1.

### 3.2. Communication Model

In this article, we consider the system with the OFDMA as the multiple access technology, in which the system bandwidth *B* is divided into *D* equal orthogonal sub-bands. In view of the OFDMA mechanism, interference is ignored due to the exclusive subcarrier allocation [25,32,33,34]. Therefore, we do not consider interference from other IoT devices in this article. A sub-band can only be allocated to one IoT device, but an IoT device can be allocated multiple sub-bands. Since the amount of data that needs to be returned to the IoT device after processing is very small, the time consumption in process of downlink transmission is not considered. Let $B_{n}$ denotes the number of sub-bandwidths allocated to IoT device *n*. $p_{n}$ denotes the transmission power of IoT device *n*. $h_{n}$ denotes the uplink channel gain between the base station and IoT device *n* corresponding to a white Gaussian noise channel, which incorporates distance based path loss model and independent Rayleigh fading. Then, the uplink transmission rate $r_{n}^{up}$ can be calculated by

(1) $$r_{n}^{up}=B_{n}\frac{B}{D}log_{2}\left(1+\frac{p_{n}h_{n}}{\delta^{2}}\right)$$

where $\delta^{2}$ denotes the noise power. Therefore, the transmission time $t_{n}^{up}$ and the energy consumption $e_{n}^{up}$ of uplink transmission can be calculated by

(2) $$t_{n}^{up}=\frac{\omega_{n}}{r_{n}^{up}}$$

(3) $$e_{n}^{up}=t_{n}^{up}\cdot p_{n}$$

### 3.3. Computation Model

In this article, the task generated by IoT device can be offload to the MEC server in order to reduce the energy consumption of the IoT device when the network is in good state. If the network state is bad, the task can only be executed on the IoT device. Next, two situations are described in detail, respectively.

#### 3.3.1. Processing at MEC Server

Let $f_{n}$ denotes the computing resources allocated by the MEC server to the task generated by IoT device *n*. Then, the execution time $t_{n}^{MEC}$ can be calculated by

(4) $$t_{n}^{MEC}=\frac{\varphi_{n}}{f_{n}}$$

#### 3.3.2. Processing at IoT Device

According to the optimization objective, if the task is processed on the IoT device, the energy consumption is the smallest when the delay is equal to the delay threshold. Therefore, the processing time $t_{n}^{IoT}$ and the energy consumption $e_{n}^{IoT}$ can be calculated by

(5) $$t_{n}^{IoT}=T$$

(6) $$e_{n}^{IoT}=k\cdot {\left(\frac{\varphi_{n}}{T}\right)}^{2}\cdot \varphi_{n}$$

where κ is the energy coefficient, which depends on the chip architecture [35,36,37]. In this article, according to the work in [38], we set $\kappa =10^{-25}$ .

## 4. Two-Timescale Joint Optimization of Task Offloading and Resource Allocation

In this section, the joint optimization of task offloading and resource allocation is formulated, and it is considered as Markov Decision Process (MDP). A deep reinforcement learning algorithm based on DDPG framework is proposed to solve this problem. In order to protect user privacy and improve the training performance of the deep neural network, Federated learning is introduced into the training model. A two-timescale federated reinforcement learning algorithm is proposed. The small timescale is to train the scheme of task offloading and resource allocation on each MEC server. The large timescale is to aggregate the trained model parameters on the central cloud server. The two-timescale are executed alternately to obtain better training performance. In this article, since the central cloud server and MEC servers are connected by the wired network, the time consumption caused by parameters upload is not considered. The detail of problem formulation and solution are described as follows.

## 4.1. Problem Formulation

According to the above computation and communication models, the total time consumption and the energy consumption can be calculated by

(7) $$t_{n}=\alpha_{n}\cdot \left(t_{n}^{up}+t_{n}^{MEC}\right)+\left(1-\alpha_{n}\right)\cdot t_{n}^{IoT}$$

(8) $$e_{n}=\alpha_{n}\cdot e_{n}^{up}+(1-\alpha_{n})\cdot e_{n}^{IoT}$$

The mathematical model with the objective of minimizing the energy consumption of all IoT devices subject to the latency requirement and the limited resources, is as follows:

(9) $$\begin{array}{c} \min_{B_{n},f_{n},\alpha_{n}}\sum_{n=1}^{N}e_{n}\\ s.t.\\ \left(c1\right)\;\;t_{n}\leq T\\ \left(c2\right)\;\;\sum_{n=1}^{N}B_{n}\leq B\\ \left(c3\right)\;\;\sum_{n=1}^{N}f_{n}\leq F^{\mathrm{MEC}}\\ \left(c4\right)\;\;\alpha_{n}\in \{0,1\} \end{array}$$

where $F^{M}EC$ where *F*^MEC^ denotes the total computing resources of the MEC server. For the constraints, constraint (*c*1) indicates that the execution time of the IoT device *n* cannot exceed the delay threshold to ensure the QoE. We believe that as long as the processing time of the IoT task is within the delay threshold, a satisfactory user experience can be obtained. For example, in the community access control system, if the delay threshold of the face recognition system is 0.1 s, the user experience can be satisfied as long as the face recognition is completed within 0.1 s. Since users have the same QoE for completing face recognition within 0.1 s and 0.01 s, there is no need to pursue a lower processing time, which is meaningless in real scenes. Constraint (*c*2) indicates that the number of allocated sub-bandwidth cannot exceed the total bandwidth of base station. Constraint (*c*3) indicates that the computing resources allocated to all IoT devices by the MEC server cannot exceed the total computing resources of the MEC server. Constraint (*c*4) indicates that the task of IoT device is either processed on the MEC server or the IoT device *n*. If $\alpha_{n}=0$ , the task of IoT device will be processed on the IoT device. If $\alpha_{n}=1$ , the task of IoT device will be offloaded to the MEC server.

## 4.2. Small Timescale Policy Based on Deep Reinforcement Learning

In this subsection, the joint optimization problem is modeled as MDP, and a deep reinforcement learning based on DDPG framework is proposed to solve it. The common model of reinforcement learning is the standard MDP. Therefore, several elements of MDP are introduced in detail below.

### 4.2.1. State Space

State is the description of the environment, which will change after an action is generated by the agent. In this article, the MEC server is modeled as an agent to optimize the energy consumption of all IoT devices. Let $s_{t}=\left(s_{t}^{1},s_{t}^{2},\ldots ,s_{t}^{U}\right)$ denotes the state of MDP at time *t*. The state includes four parts: (1) the task size, the computing workload, the channel state of all IoT devices; (2) the computing resources of the MEC server; (3) the bandwidth of the base station; (4) the resource allocation scheme at the current time. The value range of all data in the state is [0,1] .

#### 4.2.2. Action Space

Action is the description of agent behavior, which is the result of the optimization scheme. Let $a_{t}=\left(a_{t}^{1},a_{t}^{2},\ldots ,a_{t}^{L}\right)$ denotes the action of MDP at time *t*, which includes the change of computing and communication resources. The action space corresponds to Part 4 of the state space one by one. The value range of all data in the action is [−1,1] .

##### 4.2.3. Reward

After the agent takes an action, reward is the feedback of environment to agent. Let $r_{t}$ denotes the reward of MDP at time *t*. The objective of this article is to minimize the energy consumption of all IoT devices subject to the system resources and delay threshold. Therefore, the reward is set to two progressive steps. The first step is to ensure the system resources constraints, as follows:

(10) $$\begin{array}{c} r=\chi_{1}\cdot \sum_{u=1}^{U}((S^{u}-1)\cdot \varepsilon (S^{u}-1)-S^{u}\cdot \varepsilon (-S^{u}))\\ \chi_{2}\cdot (\sum_{n=1}^{N}B_{n}-B)\cdot \varepsilon (\sum_{n=1}^{N}B_{n}-B)\\ \chi_{3}\cdot (\sum_{u=1}^{U}f_{n}-F^{MEC})\cdot \varepsilon (\sum_{n=1}^{N}f_{n}-F^{MEC})+b_{1} \end{array}$$

The second step is to minimize the energy consumption of all IoT devices, as follows:

(11) $$r=\chi_{4}\cdot \mathrm{ecp}(-\sum_{n=1}^{N}e_{n}/N)$$

where $\chi_{1}$ , $\chi_{2}$ , $\chi_{3}$ , $\chi_{4}$ , $b_{1}$ are constants. The purpose is to make rewards develop in a good direction. Specifically, the reward setting algorithm is illustrated in Algorithm 1.

**Algorithm 1:** Reward calculation algorithm
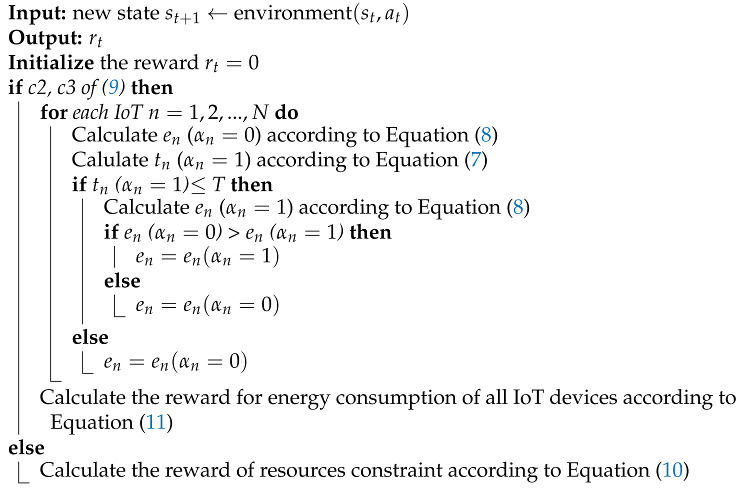



###### 4.2.4. DDPG-Based Solution

**Figure 2:**
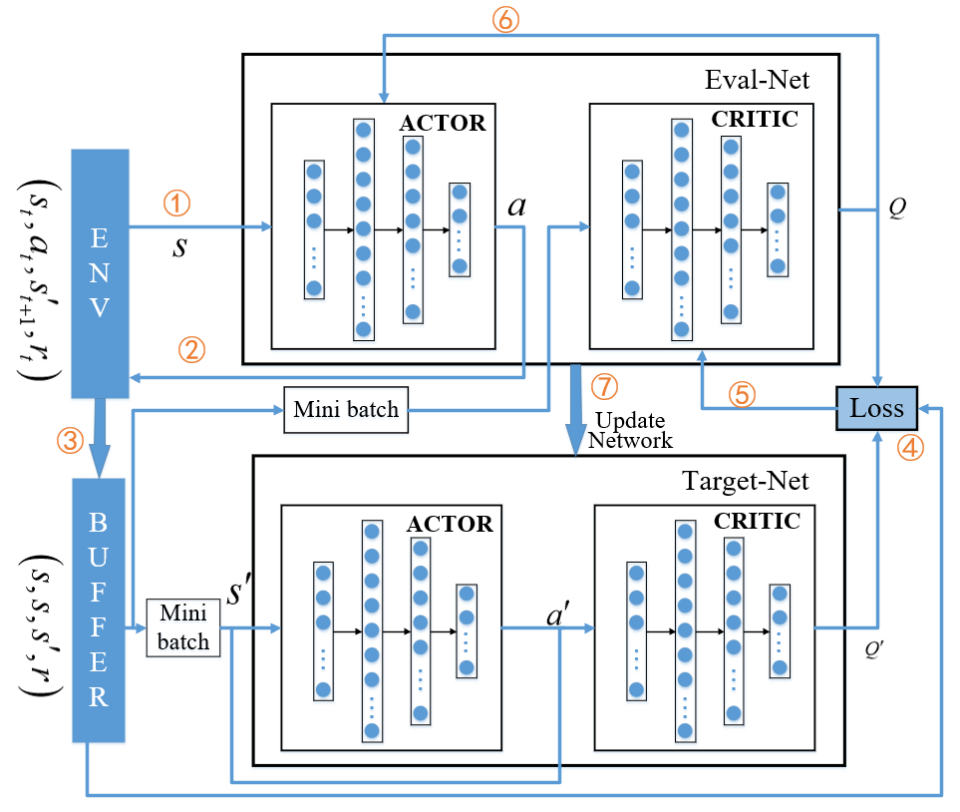
Convergence property of different algorithm.

The DDPG algorithm is a paradigm of the reinforcement learning method, which is the combination of AC and DQN. The specific network structure is shown in Figure 2. The training process of the network is carried out according to the numbers in the Figure 2. The input of the actor network is the state, and the output is the determined action value. The input of the critic network is the state and the action, the output is the *Q* value. The actor network consists of the evaluation network *µ* with parameter $\theta^{µ}$ and the target network *µ*′ with parameter $\theta^{{µ}^{\prime }}$ . The critic network consists of the evaluation network *Q* with parameter $\theta^{Q}$ and the target network $Q^{\prime }$ with parameter $\theta^{Q^{\prime }}$ . Since the experience replay method is adopted, the data $\left(s_{t},a_{t},s_{t}^{\prime },r_{t}\right)$ are stored in the replay buffer according to the format of $\left(s,a,s^{\prime },r\right)$ . The parameters of critic network are updated by minimizing the loss,

(12) $$Loss=\frac{1}{X}\sum_{j=1}^{X}{\left(y_{j}-Q\left(s_{j},a_{j}|\theta^{Q}\right)\right)}^{2}$$

(13) $$y_{j}=r_{j}+\gamma \cdot Q^{\prime }(s_{j}^{\prime },{µ}^{\prime }(s_{j}^{\prime }|Q^{\mathrm{µ\prime}})|Q^{\mathrm{Q\prime}})$$

where *X* denotes the size of mini batch data, and γ denotes the discount factor. The actor network is updated according to the feedback of the critic network as follows:

(14) $$\begin{array}{c} \nabla_{\theta }J\approx \\ \frac{1}{X}\sum_{j=1}^{X}(\nabla_{a}Q\left(s_{j},a_{j}|\theta^{Q}\right){|}_{a_{j}=\mu \left(s_{j}\right)}\cdot \nabla_{\theta µ}µ(s_{j}|\theta^{µ})) \end{array}$$

DDPG framework has the characteristics of centralized training and decentralized execution. After the training is completed, the state is input into the actor network to obtain the offloading decision and resource allocation scheme.

####### 4.2.5. Computational Complexity Analysis

Floating Point Operations (FLOPs) can be used to measure the computational complexity of the algorithm or model. The proposed algorithm is a reinforcement learning algorithm based on DDPG framework. The DDPG framework consists of an actor network and a critic network. In this article, the actor network is composed of three full connection layers, and the critic network is composed of four full connection layers. The FLOPs of a full connection layer is 2×I×Q , where *I* denotes the number of input neurons and *Q* denotes the number of output neurons. Therefore, the FLOPs of the actor network is $\sum_{m=1}^{3}2\times I_{m}\times Q_{m}$ , and the FLOPs of the critic network is $\sum_{m=1}^{4}2\times I_{m}\times Q_{m}$ . Since DDPG has the characteristics of centralized training and decentralized execution, whether the proposed framework can be implemented in a real time manner depends on the execution time of the actor network. For example, for a single core computer (2 GHz), its computing capacity is about 2 billion FLOPs per second, which is more than enough to be used to process the computation of the actor network according to network settings in this article. The specific network parameters are set in Section 5.1. Therefore, the proposed framework can be implemented in a real time manner.

## 4.3. Large Timescale Policy Based on Federated Learning

In this subsection, for the purpose of protecting privacy and security, users are reluctant to send their data the central cloud server. However, in the process of neural network training, more data will generally bring better training performance. For the above two reasons, Federated Learning algorithm is introduced into reinforcement learning. Federated learning is essentially a distributed machine learning technology. Its goal is to realize joint modeling and improve the performance of Artificial Intelligence (AI) model on the basis of ensuring data privacy, security and legal compliance. Since different blocks of smart city have the characteristic of the same application types and different users, the horizontal federated learning is adopted in this article.

In horizontal federated learning, it can be regarded as a distributed training model based on samples, which distributes all data to different machines. Each machine downloads the model from the central server to train the model with local data, and then the training parameters are returned to the central server for aggregation. In this process, each machine is the same and complete model, which can work independently. The aggregation mode of network parameters is given by

(15) $$\Theta =\frac{1}{\sum_{k\in K}D_{k}}\sum_{i=k}^{K}D_{k}\Theta_{k}$$

where $D_{k}$ denotes the number of training samples on the *k*-th MEC server, $\Theta_{k}$ and Θ denote the parameter sets of the *k*-th MEC server and the central cloud, respectively. Specifically, the two-timescale training process is summarized in Algorithm 2.

**Algorithm 2:** Training process

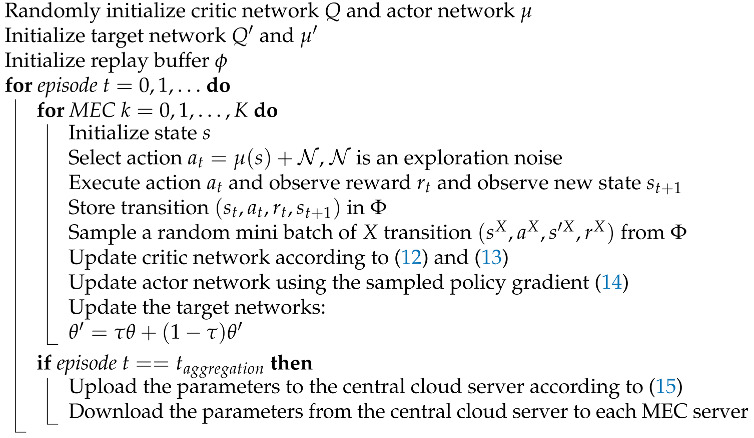



## 5. Performance Evaluation

### 5.1. Parameter Setting

In this section, we evaluate the performance of our proposed algorithm for smart city. The experimental platform adopts DELL PowerEdge (DELL-R940XA, 4*GOLD-5117, RTX2080Ti). The simulation software is Pycharm (Professional Edition). The corresponding environment configuration is Python3.7.6, CUDA 11.4, Pytorch 1.5.0. The actor network is composed of three full connection layers (40 × 500, 500 × 128, 128 × 20), and the critic network is composed of four full connection layers (60 × 1024, 1024 × 512, 512 × 300, 300 × 1). Its activation function is RELU, and the output layer of actor network is tanh function to constraint the output value. Specifically, the simulation parameters of the system are presented in Table 2. The compared algorithms are as follows.

- Random Offload:the offloading scheme of each IoT device is determined randomly. If the task of IoT device is offloaded to the MEC server, the computing and communication resources are allocated according to the proportion of data size and computing workload, respectively. If the task is executed on the IoT device, the computing resource is allocated according to the delay threshold.
- Greedy: the task of the IoT device with good channel status is offloaded to the MEC server sequentially. Each IoT device occupies the least resources to ensure that more tasks can be offloaded to the MEC server subject to the delay threshold.
- DQN: DQN is a combination of Q-learning and deep neural network, which is used to deal with the discrete state and action problem. To solve the problem in this article, the continuous state space and action space need to be discretized [9,28].
- DDPG: DDPG is the basic algorithm of this article. It is a continuous reinforcement learning algorithm, which is composed of DQN and AC.
- FL-DDPG: FL-DDPG is an algorithm proposed in this article. Federated learning is introduced into reinforcement learning to solve the problem of resource allocation and offloading decision. Since FL-DDPG has the distributed characteristic, it can improve training performance while ensuring privacy and security.

### 5.2. Convergence Analysis

**Figure 3:**
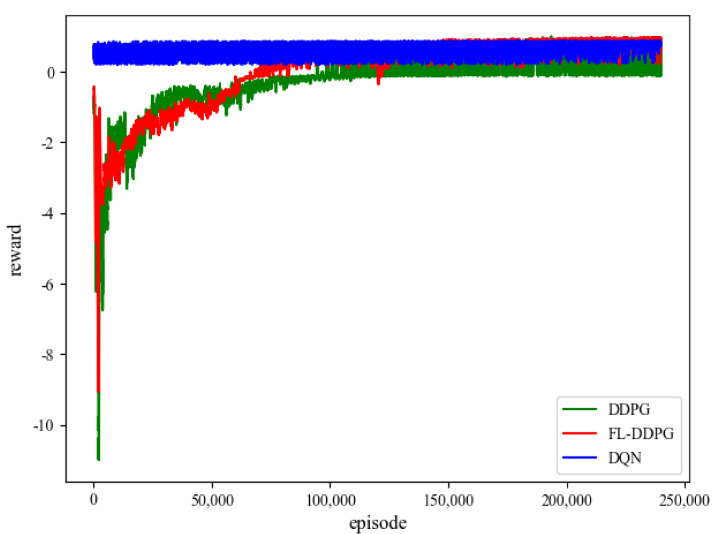
Convergence property of different algorithm.

**Figure 4:**
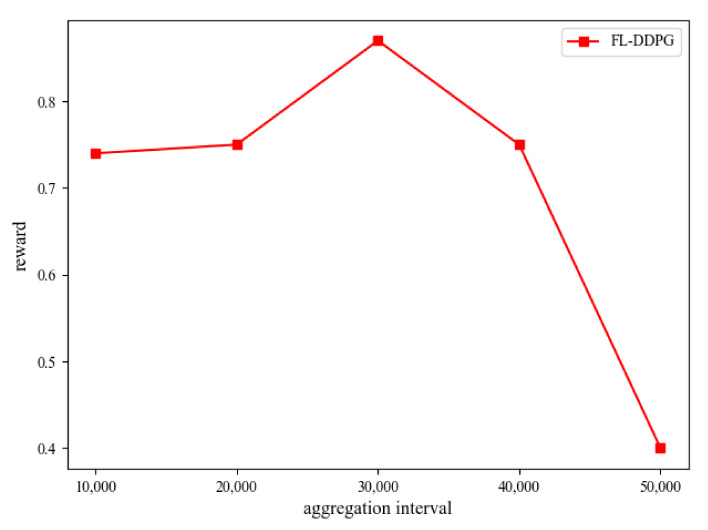
Performance evaluation on aggregation interval.

In this subsection, the convergence performance of FL-DDPG and DDPG is shown in Figure 3. In this article, the Adam optimizer is adopted to train the FL-DDPG and DDPG networks. In the training process of FL-DDPG, it needs about 240,000 training episodes (3 h) to achieve a better convergence performance. From Figure 3, we observe that the convergence performance of FL-DDPG is better than that of DDPG. Since FL-DDPG aggregates the parameters of three MEC servers, it is easy to jump out of the local optimal solution. DQN algorithm discretizes the resources, and decides where each resource block should be allocated. Therefore, DQN algorithm has no resource allocation constraints (the allocated resources will never exceed the total resources), and directly pursues the minimization of energy consumption. The value range of the reward of DQN algorithm is 0<r<1 . Since DQN algorithm is a coarse-grained resource allocation scheme, the convergence performance of DDPG is better than that of DQN. Figure 4 shows the training performance of different aggregation intervals in FL-DDPG algorithm. From Figure 4, it is observed that the training performance is the best when the aggregation interval is 30,000. When the aggregation interval is less or greater than 30,000, the training performance is not good. The reason for this is that there is not enough time to explore new environment when the aggregation interval is smaller. When the aggregation interval is larger, over-fitting is caused by too long exploration time. Therefore, the aggregation interval of 30,000 is adopted to train the network parameters in this article.

### 5.3. Performance Comparison

**Figure 5:**
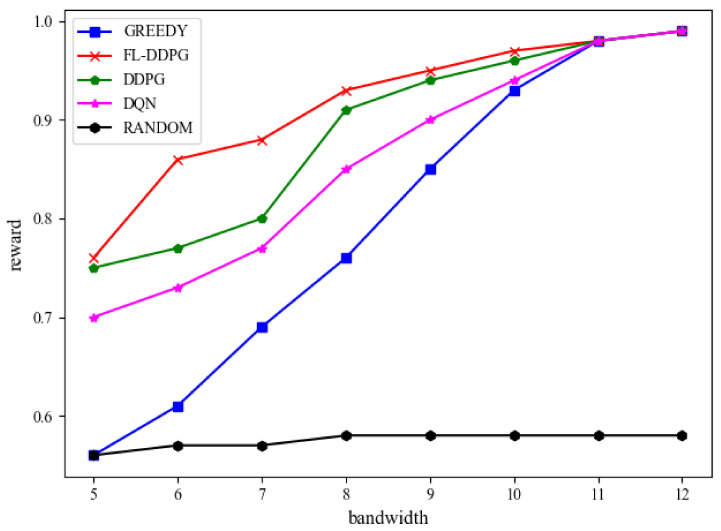
Performance evaluation on reward.

**Figure 6:**
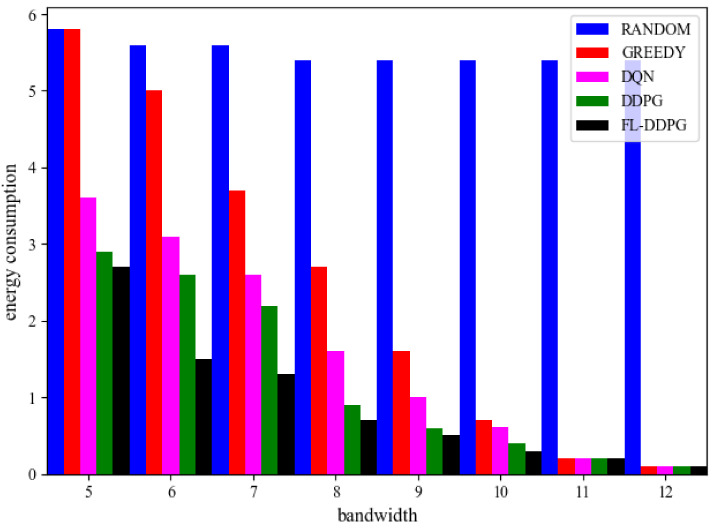
Performance evaluation on energy consumption.

**Figure 7:**
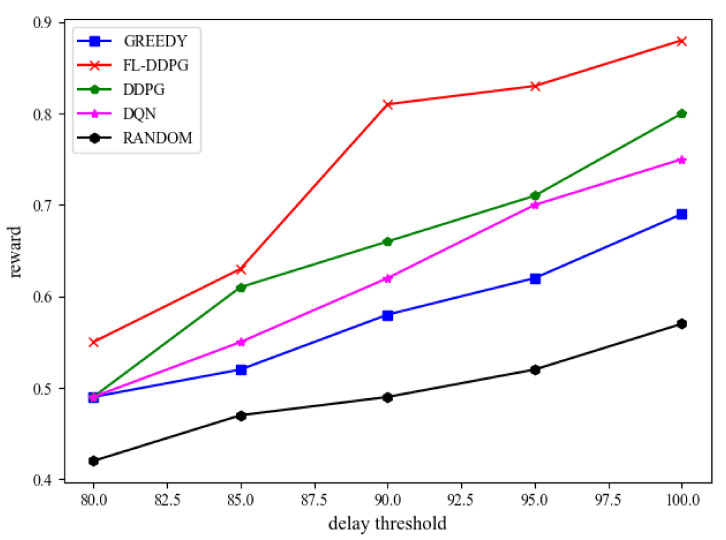
Performance evaluation on reward when the delay threshold is different.

In this subsection, the performance evaluation of different algorithms is shown in Figure 5, Figure 6 and Figure 7. Figure 5 shows the reward of different algorithms in terms of the system bandwidth. As the system bandwidth increases, more and more IoT tasks can be offloaded to the MEC server. Therefore, the energy consumption of IoT devices is reduced and the reward is increased in Figure 5. We can observe that the reward of FL-DDPG is higher than that of other algorithms. The reason can be obtained by analyzing each algorithm in detail, which is as follows. The DDPG algorithm only adopts one network model to train the decision-making scheme, which is easy to fall into local solution. Compared with DDPG and FL-DDPG, the DQN algorithm discretizes resources, which is a coarse-grained resource allocation scheme. Since DDPG algorithm is a fine-grained resource allocation scheme, the performance of DDPG algorithm is better than DQN algorithm. The GREEDY algorithm offloads the tasks generated by IoT devices with good network status to the MEC server. The algorithm only optimizes the communication resources, does not jointly optimize the communication resources and computing resources. The RANDOM algorithm is to randomly offload the tasks generated by IoT devices to the MEC server for execution. Further, we can observe that there is a little performance difference between DDPG and FL-DDPG algorithms when the system bandwidth is 5, 9, 10, 11 and 12. The reward of FL-DDPG is 1.3%, 1.1% and 1% higher than that of DDPG when the system bandwidth is 5, 9 and 10, respectively. The reason is that when the system bandwidth is very small, most of the tasks generated by IoT devices cannot be offloaded to the MEC server and can only be processed on the IoT devices. Since processing tasks on IoT devices do not involve the allocation of MEC computing resources and communication resources, the decision-making environment is simplified. Moreover, the energy consumption caused by a large number of IoT devices processing will drown out the energy consumption of transmission caused by offloading. When the system bandwidth is larger, most of the tasks generated by IoT devices can be offloaded to the MEC server. The reason for this is the same as above. Therefore, when the resources are in extreme situations, the exploration environment of reinforcement learning becomes relatively simple, resulting in a little performance difference between DDPG and FL-DDPG. In actual equipment deployment, these two extreme situations are generally not selected in terms of the cost and the quality of service. There is a large performance difference between DDPG and FL-DDPG algorithms when the system bandwidth is 6 and 7. The reward of FL-DDPG is 12% and 10% higher than that of DDPG when the system bandwidth is 6 and 7, respectively. When the system bandwidth is moderate, the decision-making environment becomes complex. The more complex the decision-making environment is, the greater the probability of DDPG algorithm falling into the local optimal solution is. Since the FL-DDPG algorithm aggregates the training parameters of three network models, it is easy to jump out of the local optimal solution.

Figure 6 shows the mean energy consumption of different algorithms in terms of the system bandwidth. From Figure 6, it is observed that the mean energy consumption of FL-DDPG algorithm is less than other algorithms. In the setting of reward, there is a negative exponential relationship between energy consumption and reward. Therefore, Figure 6 and Figure 5 are one-to-one correspondence.

**Figure 8:**
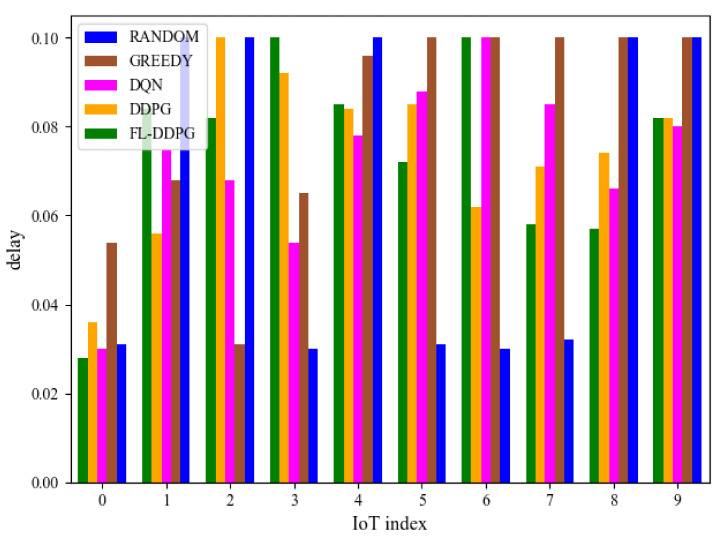
Delay of different algorithms.

Figure 7 shows the reward of different algorithms in terms of the delay threshold. In this article, since these tasks generated by IoT devices are not very strict on the response time, the delay threshold is set to the same. From Figure 7, it is observed that the reward increases when the delay threshold increases. The reason is that when the delay threshold increases, more tasks can be offloaded to the MEC server and completed within the delay threshold. Therefore, the energy consumption of IoT devices is reduced and the reward is increased. Figure 8 shows the delay of different algorithms in the same environment configuration. The delay of five algorithms is less than the delay threshold (0.1 s).

### 5.4. Analysis of Offload Location

**Figure 9:**
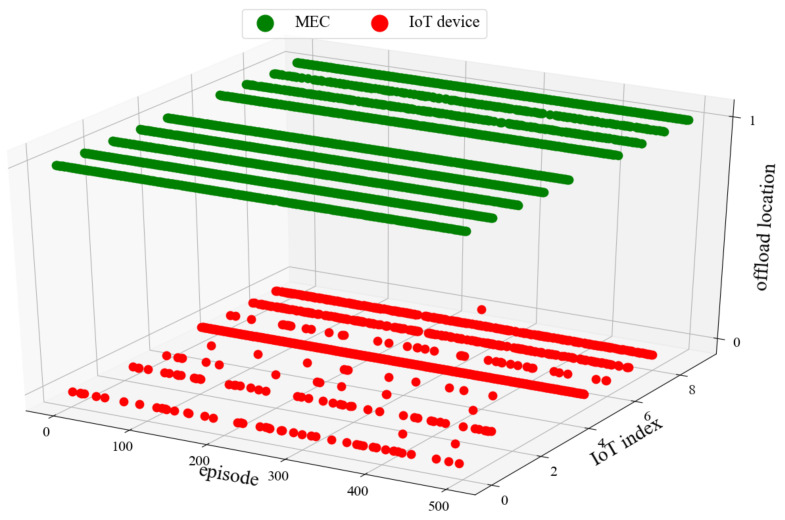
System bandwidth *B* = 5 MHz.

**Figure 10:**
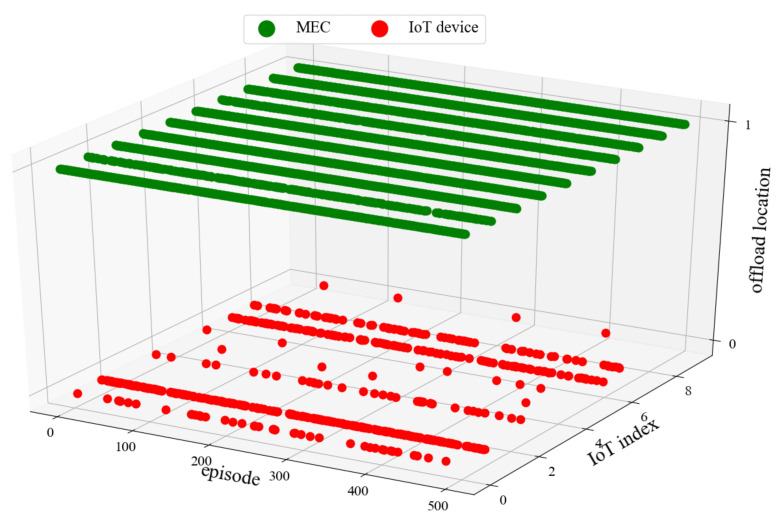
System bandwidth *B* = 10 MHz.

Figure 9 and Figure 10 show the offloading location of FL-DDPG when the system bandwidth is 5 MHz and 10 MHz. In this experiment, the X-axis denotes the number of episodes, the Y-axis denotes the IoT device index, and the Z-axis denotes the offloading location. The value range of the offloading location is 0, 1. Value 0 indicates that the task is processed on the IoT device, value 1 indicates that the task is offloaded to the MEC server. From Figure 9 and Figure 10, it is observed that the number of red points is less when the system bandwidth is 10 MHz. Figure 9 and Figure 10 indicate that more tasks are offloaded to the MEC server when the system bandwidth increases. From Figure 9, we can observe that all tasks of IoT device 6 are not offloaded to the MEC server when the system bandwidth is 5 MHz. The reason is that the task of IoT device 6 has the characteristics of large amounts of data and low computing workload. If the task of IoT devices 6 is offloaded to the MEC server, it will consume a lot of bandwidth and a small amount of the MEC computing resources. Obviously, in the case of limited resources, it is unreasonable to offload the task to the MEC server. Therefore, all tasks of IoT device 6 are processed on the IoT device.

## 6. Conclusions

In this article, a joint optimization problem of task offloading and resource allocation based on privacy protection for smart city is formulated to minimize the energy consumption of all IoT devices. First, the deep reinforcement learning algorithm based on DDPG framework is proposed to solve the mixed-integer nonlinear programming problem. Then, in order to protect user privacy and improve training performance, the federated learning is introduced into the DDPG framework. To this end, the two-timescale FL-DDPG algorithm is proposed to optimize the above problem. Specifically, the small timescale is to train the DDPG network and the large timescale is to aggregate the parameters of DDPG network. In this way, the privacy of users is not only protected, but also the performance of the algorithm is improved. We provide numerical simulation results in terms of the convergence property, reward, and energy consumption, which shows that our proposed algorithm has better performance.

## Figures and Tables

**Table 1:** Parameter descriptions.

Notation	Definition
Γ	Central cloud
*k*	Index of MEC server
*n*	Index of IoT device
$\omega_{n}$	Data size of IoT device *n*
$\varphi_{n}$	Total computing workload of IoT device *n*
$\eta_{n}$	Computing workload of IoT device *n* per bit
$\alpha_{n}$	Offloading decision of IoT device *n*
$B_{n}$	Number of sub-bandwidth allocated to IoT device *n*
*B*	System bandwidth
*D*	Number of sub-bands
$h_{n}$	Uplink channel gain between the base station and IoT device *n*
$p_{n}$	Transmission power of IoT device *n*
$f_{n}$	Computing resources allocated by the MEC server to IoT device *n*
*T*	Delay threshold of all IoT device

**Table 2:** Parameter descriptions.

Parameter	Value
Number of IoT devices	30
Number of base stations	3
Number of MEC servers	3
Uplink/Downlink system Bandwidth	10 MHz
Transmission powers of user terminal	1 W
Noise power	−100 dB
Size of task	[5, 90] Kb
Computing workload density	[200, 700] CPU cycles/bit
Path loss model	PL=127+30log(dis)
Computing resources of local device	[2, 2.5] GHz
Computing resources of MEC server	15 GHz
Delay threshold of IoT task	100 ms
episode	240,000
Mini batch	100
Buffer size	20,000
Critic network learning rate	0.001
Actor network learning rate	0.0001
Optimizer	Adam

## Data Availability

Data available on request from the authors.

## Abbreviations

The following abbreviations are used in this manuscript:

MEC	Mobile Edge Computing
IoT	Internet of Things
DDPG	Deep Deterministic Policy Gradient
FL-DDPG	Federated learning-Deep Deterministic Policy Gradient
OFDMA	Orthogonal Frequency Division Multiple Access
QoE	Quality of Experience
QoS	Quality of Service
DQN	Deep Q Network
AC	Actor-Critic
MDP	Markov Decision Process
FLOPs	Floating Point Operations
AI	Artificial Intelligence
